# *In Vitro* Characterization of Valproic Acid, ATRA, and Cytarabine Used for Disease-Stabilization in Human Acute Myeloid Leukemia: Antiproliferative Effects of Drugs on Endothelial and Osteoblastic Cells and Altered Release of Angioregulatory Mediators by Endothelial Cells

**DOI:** 10.1155/2014/143479

**Published:** 2014-01-08

**Authors:** Hilde Kvestad, Lasse Evensen, James B. Lorens, Øystein Bruserud, Kimberley J. Hatfield

**Affiliations:** ^1^Department of Clinical Science, Hematology Section, University of Bergen, 5021 Bergen, Norway; ^2^Department of Molecular Biosciences, University of Oslo, 0316 Oslo, Norway; ^3^Department of Biomedicine, University of Bergen, Norway; ^4^Department of Medicine, Haukeland University Hospital, 5021 Bergen, Norway

## Abstract

The combined use of the histone deacetylase inhibitor valproic acid (VPA), the retinoic acid receptor-**α** agonist all-trans retinoic acid (ATRA), and the deoxyribonucleic acid polymerase-**α** inhibitor cytarabine (Ara-C) is now considered for disease-stabilizing treatment of acute myeloid leukemia (AML). Leukemogenesis and leukemia cell chemoresistance seem to be supported by neighbouring stromal cells in the bone marrow, and we have therefore investigated the effects of these drugs on primary human endothelial cells and the osteoblastic Cal72 cell line. The results show that VPA and Ara-C have antiproliferative effects, and the antiproliferative/cytotoxic effect of Ara-C was seen at low concentrations corresponding to serum levels found during low-dose *in vivo* treatment. Furthermore, in functional assays of endothelial migration and tube formation VPA elicited an antiangiogenic effect, whereas ATRA elicited a proangiogenic effect. Finally, VPA and ATRA altered the endothelial cell release of angiogenic mediators; ATRA increased levels of CXCL8, PDGF-AA, and VEGF-D, while VPA decreased VEGF-D and PDGF-AA/BB levels and both drugs reduced MMP-2 levels. Several of these mediators can enhance AML cell proliferation and/or are involved in AML-induced bone marrow angiogenesis, and direct pharmacological effects on stromal cells may thus indirectly contribute to the overall antileukemic activity of this triple drug combination.

## 1. Introduction 

Acute myeloid leukemia (AML) is an aggressive bone marrow malignancy and several studies have demonstrated that different types of bone marrow stromal cells support leukemogenesis, including the maintenance of leukemic stem/progenitor cells in osteoblast-containing endosteal niches and in endothelium-containing vascular niches in the bone marrow [1, 2]. Studies of antileukemic drugs mainly focus on the pharmacological effects on the AML cell populations whereas pharmacological effects on the AML-supporting stromal cells are not so well characterized, especially not in studies of the low-toxicity disease-stabilizing therapeutic alternatives [3–6]. Several clinical studies have described an AML-stabilizing effect of valproic acid (VPA) in combination with all-trans retinoic acid (ATRA) and eventually cytotoxic drugs (e.g., Ara-C) [6–14]. VPA is a short-chain fatty acid that has multiple anticancer actions including HDAC inhibitory activity and can affect AML cell proliferation [15, 16], whereby ATRA is a vitamin A derivative that mainly interferes with regulation of differentiation and apoptosis in AML [17, 18]. Previous studies have demonstrated that all three drugs have direct effects on primary human AML cells [16, 17, 19]. Furthermore, previous *in vitro* studies have characterized the cytokine-mediated crosstalk between AML cells and neighbouring stromal cells (i.e., osteoblasts and endothelial cells) [20, 21]. This bidirectional leukemia/stromal crosstalk increased AML cell proliferation and could also affect the stromal cells [21, 22]; pharmacological targeting of AML would therefore be expected to indirectly affect the stromal cells, but antileukemic chemotherapy may also have additional direct effects on the stromal compartment that indirectly affect the leukemic cells and thereby contribute to the overall antileukemic activity. Such additional direct effects on stromal cells were recently described for pharmacological inhibition of the PI3K-Akt-mTOR pathway [23].

In the present study, we used *in vitro* experimental models to investigate how VPA, ATRA, and Ara-C directly affect endothelial cells and osteoblasts. The present results show that both VPA and Ara-C had antiproliferative effects on both stromal cell types, while ATRA did not significantly affect cell proliferation. Our functional assays of endothelial migration and capillary-like tube formation showed that VPA elicited an antiangiogenic effect *in vitro* whereas ATRA had a slightly proangiogenic effect. In addition, ATRA and VPA affected endothelial cell release of several factors that are involved in regulation of angiogenesis and/or can mediate a growth-enhancing effect on primary human AML cells. Altogether, our current results suggest that pharmacological effects of VPA/ATRA/cytarabine on stromal cells should be further investigated during clinical treatment as inhibition of stromal cell activity may potentially contribute to the overall antileukemic activity via alteration of growth factors involved in AML cell proliferation and bone marrow angiogenesis.

## 2. Materials and Methods 

### 2.1. Pharmacological Agents and Culture Medium

#### 2.1.1. Pharmacological Agents

VPA (Desitin Pharma AS, Hamburg, Germany) was purchased as a dissolved salt solution. ATRA (Roche, Oslo, Norway) powder was dissolved in ethanol. Cytosine *β*-D-arabinofuranoside (Ara-C) was purchased from Sigma-Aldrich and dissolved in sterile water. Stock solutions of each drug were made and kept stored at −80°C. Control cultures for comparisons with VPA and Ara-C treated cultures were added a solution of 0.9% NaCl, while ethanol was added to control cultures for ATRA.

#### 2.1.2. Culture Medium

We have previously investigated the effects of endothelial cells and osteoblasts on AML cell proliferation [20, 24], and the functional *in vitro* characteristics of Cal72 osteoblastic sarcoma cells were also investigated in detail in a previous study where the cells were cultured in various media [25]. The culture media used in the present study for Cal72 and endothelial cells are the same media used in our previous coculture studies. HUVECs were therefore cultured in EGM-2 medium (Lonza), while Cal72 was grown in Stem Span SFEM culture medium (referred to as StemSpan; Stem Cell Technologies, Vancouver, BC, Canada) supplemented with 10% heat inactivated fetal calf serum (FCS) (BioWhittaker, Verviers, Belgium) and 100 *μ*g/mL of gentamicin.

### 2.2. Cells and Culture Conditions

Human umbilical vein endothelial cells (HUVECs) and pulmonary artery smooth muscle cells (Pa-vSMC) were purchased from Lonza (cat no. C2517A and CC-2581, Verviers, Belgium), and the human osteosarcoma cell line Cal72 was purchased from Deutsche Sammlung von Mikroorganismen und Zellkulturen (DSMZ, cat no. ACC439, Braunschweig, Germany). Cal72 is more closely related to normal osteoblasts than other characterized osteosarcoma cells [26]. Unless otherwise stated, cells were maintained in a humidified atmosphere of 5% CO_2_ at 37°C. Cultures reaching 70–80% confluence were subcultured with a trypsin-EDTA solution (Lonza). Cell viability was evaluated by counting Trypan blue dye-excluding cells.

### 2.3. Proliferation Assays

#### 2.3.1. ^3^H-Thymidine Assay

Cells were cultured with or without drugs in flat-bottomed 96-well plates (NucleonTM Surface, Nunc A/S, Roskilde, Denmark). HUVECs were seeded at 2 × 10^3^ cells per well and Cal72 cells at 4 × 10^3^ cells per well in a total volume of 150 *μ*L per well in their respective medium. Drugs were added 18 h after seeding, and cultures were incubated for 2 days before addition of ^3^H-thymidine in 20 *μ*L of 0.9% NaCl solution (37 kBq per well, TRA 310, Amersham, UK). After 24 h, cells were transferred to a UniFilter (−96, GF/C; PerkinElmer Inc., Wellesley, MA, USA), Microscint scintillation fluid was added (PerkinElmer Inc.), and nuclear radioactivity was assayed (TopCount NXT; Packard BioScience/PerkinElmer, Wellesley, MA, USA). Only cpm values above 1000 were considered as detectable proliferation after ^3^H-thymidine incorporation. All experiments were performed in triplicate. In certain experiments, cells were cultured at low oxygen levels. Cell cultures were then placed in a multigas incubator (MCO-175M, Sanyo, Electric Co., Ltd, Tokyo, Japan) which was flushed with 5% CO_2_ and 94% N_2_. Cells were treated with drugs and incubated either at standard conditions (21% O_2_) or at 1% O_2_ before the proliferative rates were assessed.

#### 2.3.2. Cell Counting

Endothelial cells were seeded at 1 × 10^4^ cells/cm^2^ into 6-well plates (TPP, Trasadingen, Switzerland). After drug treatment for 3 days, viable cells were discriminated from dead cells by microscopy using a 0.4% Trypan Blue dye solution (Invitrogen).

### 2.4. Culture Supernatants and Cytokine Analysis

Cell-free supernatants were collected from HUVECs cultured in 6-well plates (TPP, seeding density of 1 × 10^4^ cells/cm^2^) incubated with or without drugs for 3 or 5 days. Supernatants were stored frozen at −20°C until analysis. The multiplex analysis kit (Angiogenesis Custom Prefix kits, R&D Systems) was used to measure the following ten analytes in supernatants, VEGF, VEGF-D, PIGF, bFGF, PDGF-AA, PDGF-BB, angiopoietin-1 (Ang-1), angiogenin, endostatin, and thrombospondin-2. Concentrations of CXCL8, CXCL10, MMP-2, sVEGFR1, sVEGFR2, and HGF in supernatants were determined in duplicates using Quantikine enzyme-linked immunosorbent assays (ELISA) (R&D Systems, Minneapolis, MN, USA).

### 2.5. Endothelial Migration Assay

HUVECs were seeded into 6-well plates and preincubated with VPA (0.3–2.4 mM) or ATRA (0.01 *μ*M–1 *μ*M) for 3 days, then harvested and washed before being resuspended in EBM-2 media (basal medium without added growth factors or serum). A total of 5 × 10^4^ viable cells in 0.2 mL EBM-2 were seeded into 8 *μ*m pore inserts in 24-well plates (Falcon), and the lower chamber was added 20% FBS in 0.5 mL EBM-2 as a chemoattractant. After 6 h of incubation, cells on the upper side of the porous membrane were removed, while migrated cells on the lower membrane surface were fixed with paraformaldehyde and stained with 5 *μ*g/mL Hoechst 33342 for nuclei visualization. For each membrane, four fields of view were imaged using a 10x objective on a Zeiss AxioObserver microscope. ImageJ software (NIH Image, NIH) was used to quantify the number of migrated endothelial cells.

### 2.6. Endothelial Capillary-Like Network Formation Assay

We used the *in vitro* coculture angiogenesis assay as described in detail previously [27, 28]. Briefly, early passage HUVECs were infected with retrovirus carrying a fluorescent (GFP)—expressing construct. HUVECs and Pa-vSMCs were simultaneously seeded into half-area 96-well plates (cat. no. 675090; Greiner Bio-One, Essen, Germany); plates were then centrifuged at 200 g and incubated for 4 h to allow cell attachment before addition of drugs (50 *μ*L per well, total volume per well 150 *μ*L). Cells were seeded into complete EGM-2 when testing for antiangiogenic activity, while EBM-2 medium supplemented with only hydrocortisone, ascorbic acid, gentamicin, heparin, and 2% FBS (all from the EGM-2 bullet kit) was used when testing for potential proangiogenic activity of drugs [29]. Cocultures were automatically imaged using the BD Pathway 855 high-throughput BioImager (BD Biosciences, San Jose, CA). Images were acquired as 2 × 2 montages to enlarge the view field using a 10x objective, with an excitation filter 488/10 and emission filter 520/35 to visualize GFP-expressing cells. Image-based autofocus ensured to bring cells into focus in every well to obtain high quality images.

### 2.7. Statistical Analyses

All data were analyzed using GraphPad (GraphPad Prism 5 Software, CA, USA). Standard error of the mean (SEM) is shown when at least three independent experiments are performed, and one-way ANOVA with Tukey's multiple test was used for multiple comparisons between treatments (expressed as percentage of control values). The inhibitory concentration of 50% (IC_50_) was calculated as the concentration of drug yielding 50% reduction compared with untreated control.

## 3. Results

### 3.1. Effects of VPA, ATRA, and Cytarabine on Stromal Cell Proliferation: A Comparison of HUVEC and Osteoblastic Cal72 Cells

**Figure 1:**
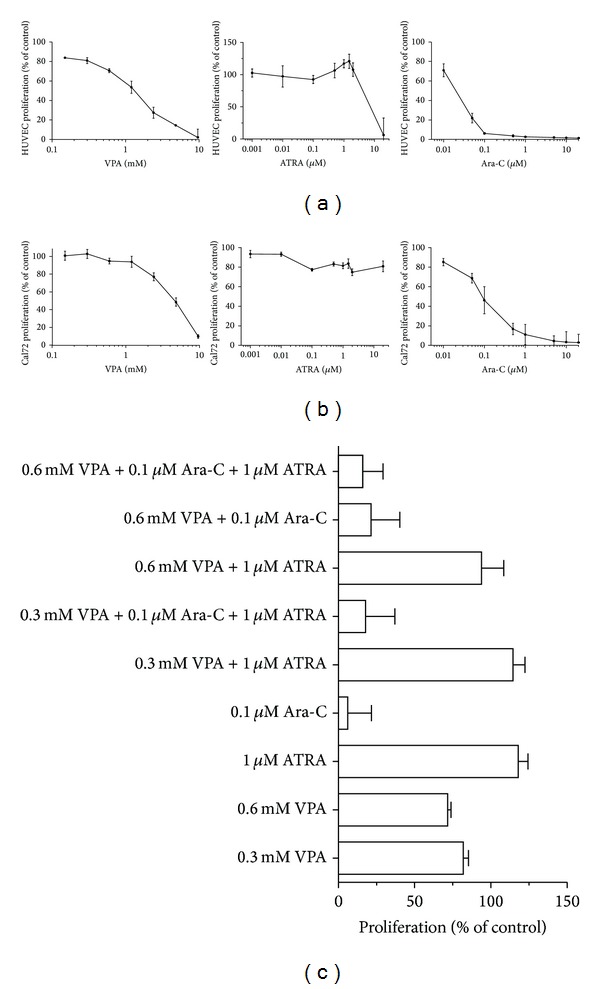
Effects of VPA, ATRA, and Ara-C on stromal cell proliferation. (a) HUVECs and (b) Cal72 cells were incubated with various concentrations of VPA, ATRA, or Ara-C for 3 days and proliferation was measured as incorporation of ^3^H-thymidine the last 24 hours of incubation. (c) The figure shows the effect of Ara-C (0.1 *μ*M), VPA (0.3 and 0.6 mM), and ATRA (1 *μ*M) treatment on HUVEC proliferation alone or in drug combinations compared to drug-free control cultures. Results are expressed as mean percentage ± SEM from three independent experiments (six replicates per condition) relative to corresponding untreated control cultures.

Bone marrow angiogenesis seems to be important for leukemogenesis and also chemosensitivity in human AML, and both endothelial and osteoblastic cells are able to support the proliferation of primary human AML cells [20, 24]. In this context, we investigated the effects of the AML-stabilizing drugs VPA, ATRA, and cytarabine on growth of osteoblastic Cal72 cells and HUVECs. The cells were cultured for 3 days with different concentrations of VPA (0.15–9.6 mM), ATRA (0.001–20 *μ*M), and Ara-C (0.01–20 *μ*M) before proliferation was analyzed using the ^3^H-thymidine incorporation assay. VPA and Ara-C had dose-dependent antiproliferative effects for both endothelial and osteoblastic cells (Figures 1(a) and 1(b)). ATRA 20 *μ*M caused a significant decrease of HUVEC proliferation, but ATRA did not significantly inhibit HUVEC or Cal72 proliferation when tested in the range from 0.001 *μ*M to 2 *μ*M. HUVEC cell growth was significantly inhibited by VPA concentrations ≥0.15 mM compared to drug-free control cultures, while Cal72 growth was significantly inhibited by a higher dose (2.4 mM) of VPA compared to control cultures (Tukey's multiple test, *P* < 0.05, *n* = 3). In addition, 0.01 *μ*M Ara-C significantly inhibited cell growth compared to drug-free control cultures for both HUVEC and Cal72 (Tukey's multiple test, *P* < 0.05, *n* = 3).

IC50 values for VPA in the ^3^H-thymidine proliferation assay were 1.1 mM for HUVEC and 4.8 mM for Cal72. The IC50 values obtained after Ara-C treatment were 19.1 nM for HUVECs and 95.1 nM for Cal-72. The effect of cytarabine on cell proliferation was in addition tested at two different concentrations (0.1 and 1 *μ*M) in combination with VPA (0.3 and 0.6 mM) and/or ATRA (1 *μ*M). Serum levels corresponding to these Ara-C concentrations can be reached during low-dose Ara-C therapy [30]. A similar antiproliferative effect of Ara-C was observed also in the presence of VPA as well as ATRA for both cell types, and VPA/ATRA did not alter the proliferation compared to treatment with cytarabine alone at these concentrations (Figure 1(c), results shown for HUVECs).

We also examined the effects of Ara-C combined with VPA and ATRA on stromal cell proliferation at low oxygen levels, and the proliferation of both HUVEC and Cal72 was reduced with >50% when cells were cultured in drug-free conditions at 0.1% O_2_ compared with culture at 21% O_2_. Even though growth was reduced during low oxygen levels, cpm values above 5000 were measured in drug-free cultures after 0.1% O_2_ culture, and a similar growth-reducing effect was seen for Ara-C (0.1 and 1 *μ*M) and VPA (0.3 and 0.6 mM) when both stromal types were incubated at 0.1% O_2_ compared to 21% O_2_, relative to their respective control cultures grown at the same oxygen concentrations, and VPA/ATRA did not alter the proliferation compared to treatment with Ara-C alone (data not shown). Thus, the dose-dependent antiproliferative effects of Ara-C on both HUVEC and Cal72 cells was maintained in the presence of VPA and ATRA also during culture at low oxygen levels.

### 3.2. Viable Cell Counts after Treatment of HUVECs with VPA and ATRA

**Figure 2:**
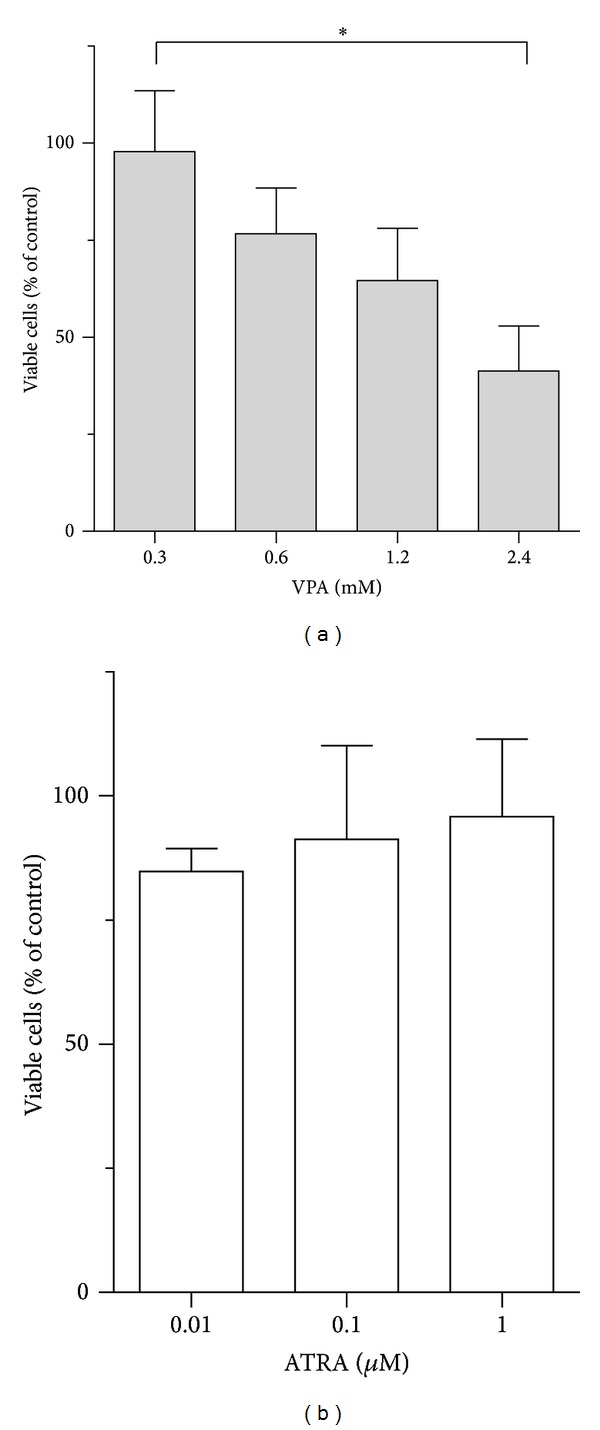
Effects of VPA and ATRA on endothelial cell viability. HUVECs were treated with VPA and ATRA for 3 days and viable cells were counted as Trypan blue dye-excluding cells. The number of cells decreased after VPA treatment in a dose-dependent manner, while ATRA did not alter the number of viable cells, and the percentage of viable cells was >80% after treatment with all doses of ATRA. Results are shown as the mean number of viable cells in percent compared to control cultures ± SEM, for three independent experiments (*P < 0.05, Tukey's multiple test).

HUVECs were cultured for 3 days in the presence of VPA or ATRA before the number of viable cells was determined by counting Trypan blue dye-excluding cells. VPA (range 0.3–2.4 nM) caused a dose-dependent reduction in the number of viable cells (*P* < 0.05, control versus 2.4 mM VPA), while ATRA (range 0.01–1 *μ*M) did not significantly alter the number of viable cells and the amount of viable cells was >80% compared to control cultures after treatment with all doses of ATRA (Figure 2). The percentage of dead cells (Trypan Blue stained) was less than 10% after treatment of all doses of VPA and ATRA. These results correspond to the cell proliferation results, where ATRA did not affect cell growth at concentrations below 2 *μ*M, though VPA inhibited cell proliferation with increasing concentrations.

### 3.3. Effects of VPA and ATRA on Migration and Capillary-Like Network Formation by HUVECs

**Figure 3:**
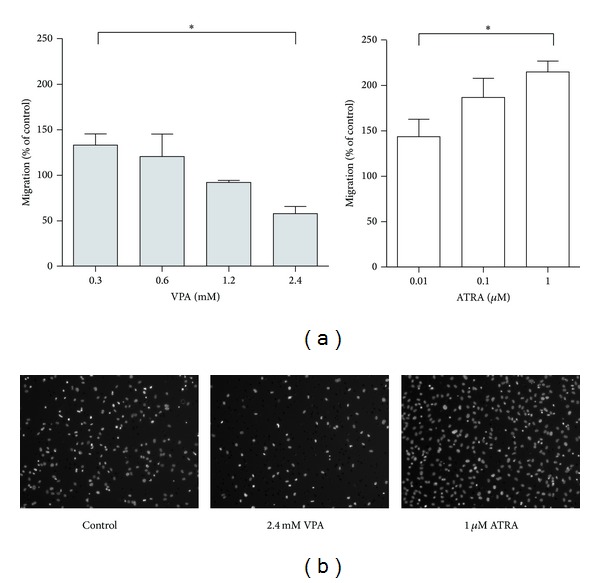
Effects of VPA and ATRA on endothelial cell migration. (a) HUVECs were treated for 3 days with VPA-concentrations ranging from 0.3 mM to 2.4 mM and ATRA-concentrations from 0.01 *μ*M to 1 *μ*M before cell migration through 8 *μ*m pore filters was determined using a transwell assay. Results are shown as mean migrated cells ± SEM (percent migrated cells compared to control cultures) for three independent experiments (*P < 0.05, Tukey's multiple test). (b) Images show Hoechst-stained migrated cells on transwell filters for the highest drug concentrations of VPA and ATRA resulting in significantly altered cell migration. A representative drug-free control image for comparison with treated cultures is shown.

AML is characterized by increased bone marrow angiogenesis [31], and this process depends on endothelial cell proliferation as well as endothelial cell migration and organization. To examine whether VPA and ATRA have effects on endothelial cell migration, cells were incubated with VPA (0.3/0.6/1.2/2.4 mM) or ATRA (0.01/0.1/1 *μ*M) for 3 days and cell migration was determined using transwell culture inserts. A dose-dependent decrease of HUVEC migration was observed after 3 days of VPA treatment (Figures 3(a) and 3(b)), while cell migration was enhanced by increasing doses of ATRA compared to the respective controls.

**Figure 4:**
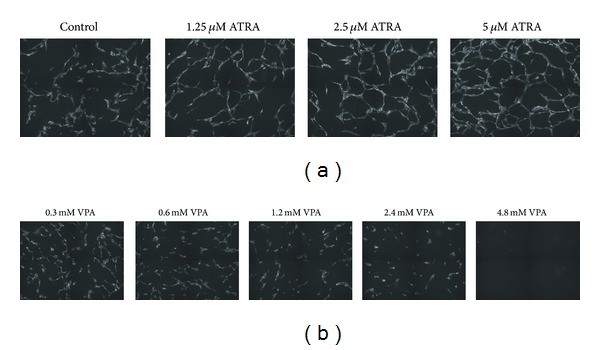
Altered endothelial cell network formation after treatment with VPA and ATRA.  Representative images acquired after 3 days of coculture in the presence of drugs show network formation after (a) treatment with ATRA and (b) treatment with VPA. A representative image of a drug-free control culture is shown for comparison with VPA and ATRA treated cultures. Pa-vSMCs form a confluent layer beneath the HUVECs, but only the fluorescent endothelial cells are visible in the images.

A coculture assay was used to investigate the effects of VPA and ATRA on endothelial cell capillary-like formation *in vitro*. HUVECs in coculture with Pa-vSMCs generate capillary-like networks in the presence of growth-factor rich endothelial culture medium (EGM-2), but neither VPA nor ATRA altered the degree of network formation when added to cocultures in this growth-factor rich medium; however, VPA showed potent inhibition of capillary-like network formation in a dose-dependent manner when added to cocultures grown in the growth factor-reduced EBM-2 medium (Figure 4(b)), whereas enhanced network formation was seen after treatment with ATRA (Figure 4(a)). Hence, we used two different assays to investigate endothelial cell migration/organization that are important steps in the process of angiogenesis; VPA then caused an inhibition of HUVEC migration/capillary-like tube formation in both assays while ATRA seemed to have an enhancing effect in these assays.

### 3.4. Effects of VPA and ATRA on the Release of Soluble Mediators by Endothelial Cells

Previous experimental studies have demonstrated that the cytokine-mediated crosstalk between primary human AML cells and endothelial cells promotes leukemia cell proliferation [20, 21, 24]. This crosstalk-dependent growth enhancement can be mediated by various cytokines including IL1, GM-CSF, VEGF, CXCL8, and HGF, but the relative contribution of each mediator seems to vary between patients [21]. Investigation of the cytokine release profile of HUVECs showed that these cells release very low or undetectable levels of IL1*β*, GM-CSF, and HGF under *in vitro* culture conditions; however, they release (i) several mediators important for angioregulation including MMP-2, endostatin, and various angioregulatory chemokines [32]; and (ii) cytokines known to promote AML cell proliferation including CXCL8 and PDGF [32, 33]. Drug-induced modulation of angioregulatory cytokine production and consequently altered communication between neighboring cells could be a possible explanation for the altered tube formation described above, and in this context we investigated the effects of VPA and ATRA on angioregulatory mediators and AML growth-enhancing factors released during culture of HUVECs. The culture medium EGM-2 was supplemented with growth factors provided by the distributor and we then measured high levels of bFGF (720 pg/mL) and VEGF (554 pg/mL) in culture medium alone, whereas low/undetectable levels of VEGF (<20 pg/mL) and bFGF (<100 pg/mL) were detected in the supernatants after culture of HUVECs for 3–5 days. High Ang-1 levels (652 pg/mL) were also found in the culture media, though levels were not altered during culture.

**Figure 5:**
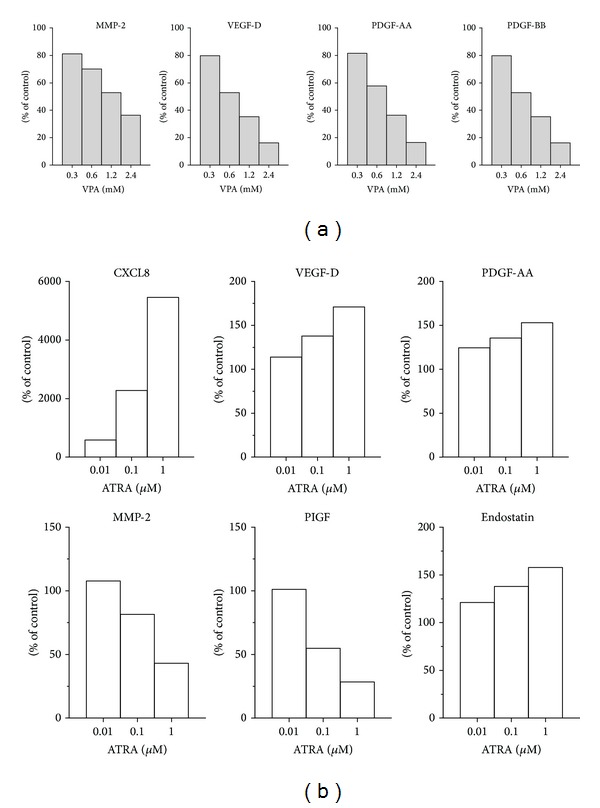
VPA and ATRA modify the release of diffusible mediators from endothelial cells. Concentrations of soluble mediators were determined in HUVEC culture supernatants after treatment with different doses of VPA or ATRA for 5 days. Colorimetric ELISA kits and a Luminex assay using multianalyte profiling beads were utilised to determine the different mediator concentrations in duplicate samples after treatment with (a) VPA and (b) ATRA. The results are presented as the mean percent alteration (duplicate samples) of mediator concentrations found in treated cultures compared with their respective drug-free control cultures containing 0.9% NaCl (VPA control) or ethanol (ATRA control).

In supernatants collected from HUVECs cultured alone for 5 days, we measured detectable release of angiogenin (2157 pg/mL), PDGF-AA (1292 pg/mL), PDGF-BB (1500 pg/mL), endostatin (5523 pg/mL), thrombospondin (169 pg/mL), PIGF (149 pg/mL) and VEGF-D (143 pg/mL), CXCL8 (368 pg/mL), MMP-2 (116 ng/mL), sVEGFR-1 (>200,000 pg/mL), and sVEGFR-2 (152 pg/mL), while HUVEC did not produce HGF or CXCL10, and detectable release of these latter two cytokines were not induced by addition of VPA or ATRA. However, VPA and ATRA modified the release of several mediators in a dose-dependent manner compared to levels found in untreated controls (Figures 5(a) and 5(b)). These dose-dependent effects were most clearly seen after 5 days compared to treatment for only 3 days, and therefore only levels measured after 5 days of treatment are shown in Figure 5. Both VPA and ATRA caused a dose-dependent decrease of MMP-2 levels. Proangiogenic CXCL8 levels increased in the presence of ATRA while no dose-dependent effect on CXCL8 levels was seen after VPA treatment. CXCL8 levels were altered the most of all the mediators examined; levels increased from 254 pg/mL in drug-free control cultures to 13,887 pg/mL (a 54-fold increase) after 1 *μ*M ATRA treatment for 5 days. Thrombospondin-2, endostatin, angiogenin, and PIGF levels were slightly reduced by VPA treatment, while unaltered levels of thrombospondin-2 and angiogenin were seen after ATRA treatment, and in addition, PDGF-BB levels were slightly increased by ATRA treatment (data not shown). HUVEC controls treated with ethanol and 0.9% NaCl had no effects on the release of mediators.

**Figure 6:**
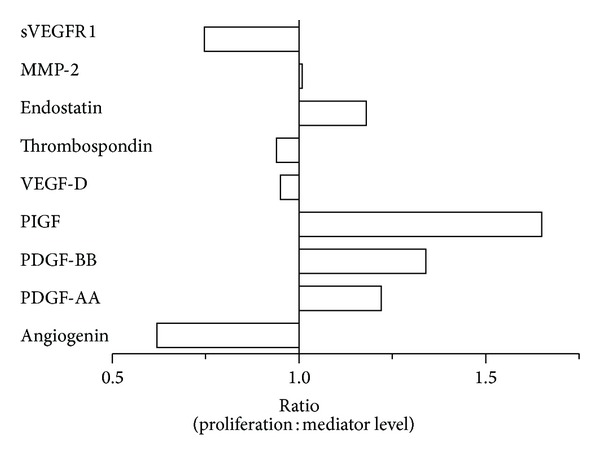
The relative ratio of cell proliferation versus mediator release from HUVECs after VPA treatment. The proliferation of HUVECs after 0.6 mM VPA treatment was reduced to 71% of drug-free controls, and the figure shows the relative ratio values of proliferation after 0.6 mM VPA treatment versus the concentration of various soluble mediators measured in HUVEC cultures after 0.6 mM VPA treatment.

We measured levels of sVEGFR1 and sVEGFR2 released by HUVEC cells, which are truncated isoforms of VEGFR1 and VEGFR2, respectively. VPA decreased levels of sVEGFR1 and sVEGFR2, while slightly increased levels were found after ATRA treatment (data not shown). In summary, mediator levels were generally decreased after VPA treatment, while divergent results were seen after ATRA treatment. As VPA had antiproliferative effects, we compared the ratio of proliferation/mediator release measured after treatment with 0.6 mM VPA to see if effects of VPA-treatment on cell proliferation may account for the reduced levels of mediators released by HUVECs. The ratio varied between the different mediators indicating that other effects besides reduced proliferation/cell numbers also contribute to modulation of endothelial mediator release (Figure 6). Taken together, these observations show that ATRA and VPA can affect endothelial cell release of several mediators that are (i) known to be involved in regulation of angiogenesis [32] and/or (ii) have a growth-enhancing effect on primary human AML cells during *in vitro* coculture of leukemic cells with endothelial cells [24].

## 4. Discussion

Stromal cells are involved in regulation of both normal and leukemic hematopoiesis [1, 2, 34, 35], and AML chemotherapy seems to have indirect antileukemia effects mediated via effects on stromal cells. Furthermore, low-toxicity disease-stabilizing treatment based on VPA, ATRA, and eventually conventional cytotoxic drugs is being considered in the treatment of human AML. In this context, we used HUVECs as a model system for *in vitro* studies of endothelial cell function after treatment with VPA, ATRA, and low concentrations of Ara-C on endothelial cells and also performed comparative growth studies using the Cal72 osteosarcoma cell line that has been used in several experimental studies and is regarded to have an osteoblastic phenotype [26]. Altogether, our study suggests that pharmacological agents used in disease-stabilizing treatment of human AML directly affect stromal cells and thereby may have indirect antileukemic activity. A possible antileukemic mechanism is then reduced release of cytokines by stromal cells that are known to mediate growth enhancement of the leukemia cells during *in vitro* coculture of primary human AML cells and endothelial cells [24].

We have previously used *in vitro* cocultures of leukemic and stromal cells to demonstrate that endothelial as well as osteoblastic cells can have a cytokine-mediated growth-enhancing effect on primary human AML cells [20, 24]. In the present study, we used the same culture conditions as used in our previous coculture studies, and we then show that VPA and Ara-C inhibit endothelial cell proliferation (also seen for osteoblastic cells), while VPA and ATRA alter endothelial cell migration and cytokine release. The experimental conditions were similar in both our previous coculture and current culture studies, and we now observed altered release of soluble mediators including both angioregulatory mediators and cytokines that can function as AML growth factors during coculture of AML and stromal cells. The drug-induced effects on HUVEC proliferation and migration/orientation may then represent a second indirect antileukemic mechanism that interferes with leukemia-induced angiogenesis.

Several clinical studies have investigated VPA in combination with other drugs for the treatment of AML [7–10, 14, 36, 37], and the concentrations of VPA and ATRA utilized in our *in vitro* assays are based on serum levels reported in these clinical studies [38]. The median serum level of VPA in one study was 360 *μ*M (range of 214–743 *μ*M) [38], but response to treatment can be seen even at lower concentrations [3]. Therefore, we chose to use a low dose of VPA 300 *μ*M, an intermediate dose of 600 *μ*M, and high doses of ≥1200 *μ*M in our assays. ATRA plasma levels have a high interpatient variability, and patients treated with ATRA were shown to have significantly decreased plasma levels after a few days of treatment and levels remained low or undetectable even after treatment with increased doses [39]. Following the usual oral dose of 45 mg/m^2^ ATRA, median peak plasma concentrations were measured to be 1 *μ*M [40]. In addition, one of the first studies of ATRA showed an effect on leukemia cell lines *in vitro* at 0.001 *μ*M, with a maximum effect on differentiation at 1 *μ*M [41], and therefore the physiological relevant level of 1 *μ*M ATRA was chosen for all our experiments.

Cytarabine is a deoxycytidine analogue [19] that is metabolized to its active triphosphate form that inhibits deoxyribonucleic acid polymerase-*α* and is incorporated into elongating DNA strands and thereby causes chain termination. The serum levels of Ara-C depend on the dose and administration of the drug: (i) conventional doses vary from 100 to 200 mg/m^2^ given by intermittent injection of continuous infusion over 5–10 days and result in steady-state plasma levels of 0.1–1 *μ*M [42]; (ii) high-dose protocols administering the drug as 1–3 g/m^2^ results in peak concentrations of 100 *μ*M (up to 300 *μ*M in children) but a rapid fall to 0.11–8.25 *μ*M [19, 43, 44]; and (iii) low-dose therapy with 10–20 mg/m^2^ once or twice daily reaches maximal levels of 0.2–0.6 *μ*M 15 minutes after the injection when using 10 mg/m^2^ [30] and this is well above 0.1 pM that is assumed to be required for cytotoxicity [19]. Furthermore, the active metabolite uracil arabinoside may show 5–8-fold higher serum levels than Ara-C and has a relatively long half-life [43]. We tested Ara-C at a concentration range from 0.01 to 20 *μ*M; the lower concentrations of 0.01–5 *μ*M are then considered relevant for low-dose cytarabine therapy and these concentrations will then result in cytotoxic activity. Our results demonstrate that the cytotoxic/antiproliferative effects of these low Ara-C levels are maintained in the presence of clinically relevant VPA and ATRA levels. Other studies have reported other IC50 values after VPA or Ara-C treatment of HUVECs than those we found in our *in vitro* proliferation assay [45, 46]; however, IC50 values may vary due to the utilization of different assays, different incubation time of drugs, and the end point concentration values used [47]. However, the observations by Michaelis et al. [45] support the clinical observations that VPA can have antileukemic effects *in vivo* at concentrations lower than the generally accepted therapeutic serum level used for antiepileptic therapy.

Our present study investigated the *in vitro* effects of VPA and ATRA on various steps in the process of angiogenesis. Previous studies have investigated how ATRA modulates angiogenesis, though the results are not clarifying as both a stimulating and inhibitory effect on angiogenesis has been shown [48–50]. We therefore investigated the effect of ATRA on endothelial cells using two different assays to examine angiogenesis. ATRA enhanced the migration of HUVECs as well as the organization of endothelial cells into capillary-like networks in a dose-dependent manner, which supports a proangiogenic effect of ATRA. In contrast, VPA inhibited the ability of HUVECs to migrate and decreased capillary-like networks in our coculture model of endothelial cells and supporting vascular smooth muscle cells that form capillary-like vessels and thereby mimics* in vivo* conditions [27]. Our present observations of an antiangiogenic effect of VPA *in vitro* are in concordance with studies of VPA and other HDAC inhibitors in other experimental models [45, 51].

In our functional *in vitro* assays, ATRA enhanced HUVEC migration and network formation and increased the levels of several proangiogenic factors, including CXCL8 and VEGF-D. One possible mechanism for increased expression may be due to ATRA-induced upregulation of HIF-1*α* expression [52]. However, ATRA also reduced levels of proangiogenic MMP-2, which concords with findings by another study [53]. These observations reflect that there is a complex balance between the collective action of proangiogenic and antiangiogenic factors which influences the final effect on the process of angiogenesis [29], and in addition, other effects (e.g., antiproliferative and proapoptotic) may not be reflected in our *in vitro* models of angiogenesis. ATRA strongly enhanced the release of angiogenic mediators including CXCL8 by the endothelial cells; this may *in vivo* either result in (i) direct effects on autocrine cell signaling, (ii) inhibition of effects that ECs have on pericytes surrounding blood vessels or other stromal cells in the bone marrow, or (iii) it may potentially have an indirect paracrine effect on leukemia cells* in vivo*. We did not see an effect of VPA on CXCL8 release, though similar therapeutically relevant VPA-concentrations have been shown to induce the mRNA expression of other members of the CXC chemokine family in HUVECs [54]. Diffusible factors secreted from endothelial cells have also been shown to have a role in maintenance of hematopoietic stem cells (HSCs) and leukemia cells [34, 35].

Even though our *in vitro* results should be interpreted carefully, our observations suggest that VPA may mediate antileukemic activity in part through inhibition of angiogenesis; this is supported by the observed antiproliferative effects on HUVEC cells, the inhibition of proangiogenic factors released by HUVECs and reduced HUVEC migration and capillary tube formation. Other studies have also shown that VPA induces G0/G1 cell cycle arrest of both HUVECs and AML cells [55, 56], which is consistent with our findings of a reduced cell number after VPA treatment and a low amount of dead cells (Trypan-blue excluding cells). Continuous VPA treatment can be combined with intermittent ATRA therapy for disease-stabilizing treatment in human AML [6, 10, 17], and it is more likely that the antiangiogenic and antiproliferative effects of VPA would then be regarded as more important than the proangiogenic effects mediated by ATRA, though further studies must be performed to address the relative importance of direct versus indirect antileukemic effects during AML-stabilizing treatment.

Altogether, our results suggest that VPA acts not only as a direct antileukemic drug, but may also exert antiproliferative and antiangiogenic activity on the stromal cell compartment that in turn may have indirect antileukemic effects. However, it is recognized that these *in vitro* studies are limited in that they are conducted apart from the leukemic microenvironment. Our present studies were performed by using the same experimental conditions as for previous coculture experiments [20, 24], and the described pharmacological effects include reduced endothelial cell release of AML growth factors that mediate the endothelium-induced growth enhancement during coculture. The effects on endothelial cell proliferation/migration/orientation may represent a second indirect antileukemic effect by these drugs through altered local angioregulation. VPA and ATRA may also be combined with other antileukemic drugs, for example, 5-mercaptopurine, hydroxyurea, and 5-azacitidine [9, 12, 57], and only future clinical studies can clarify which combination is optimal. However, ATRA/VPA/cytarabine-based treatment is effective only for a subset of patients [16, 38], and future clinical studies should also investigate whether new biomarkers can be used to identify patients that will respond to treatment.
